# A Monte Carlo simulation study comparing linear regression, beta regression, variable-dispersion beta regression and fractional logit regression at recovering average difference measures in a two sample design

**DOI:** 10.1186/1471-2288-14-14

**Published:** 2014-01-24

**Authors:** Christopher Meaney, Rahim Moineddin

**Affiliations:** 1 Department of Family and Community Medicine, University of Toronto, 500 University Avenue, Toronto M5G1V7, ON, Canada

**Keywords:** Regression modelling, Linear regression, Beta regression, Variable-dispersion beta regression, Fractional Logit regression, Beta distribution, Multinomial distribution, Monte Carlo simulation

## Abstract

**Background:**

In biomedical research, response variables are often encountered which have bounded support on the open unit interval - (0,1). Traditionally, researchers have attempted to estimate covariate effects on these types of response data using linear regression. Alternative modelling strategies may include: beta regression, variable-dispersion beta regression, and fractional logit regression models. This study employs a Monte Carlo simulation design to compare the statistical properties of the linear regression model to that of the more novel beta regression, variable-dispersion beta regression, and fractional logit regression models.

**Methods:**

In the Monte Carlo experiment we assume a simple two sample design. We assume observations are realizations of independent draws from their respective probability models. The randomly simulated draws from the various probability models are chosen to emulate average proportion/percentage/rate differences of pre-specified magnitudes. Following simulation of the experimental data we estimate average proportion/percentage/rate differences. We compare the estimators in terms of bias, variance, type-1 error and power. Estimates of Monte Carlo error associated with these quantities are provided.

**Results:**

If response data are beta distributed with constant dispersion parameters across the two samples, then all models are unbiased and have reasonable type-1 error rates and power profiles. If the response data in the two samples have different dispersion parameters, then the simple beta regression model is biased. When the sample size is small (N_0_ = N_1_ = 25) linear regression has superior type-1 error rates compared to the other models. Small sample type-1 error rates can be improved in beta regression models using bias correction/reduction methods. In the power experiments, variable-dispersion beta regression and fractional logit regression models have slightly elevated power compared to linear regression models. Similar results were observed if the response data are generated from a discrete multinomial distribution with support on (0,1).

**Conclusions:**

The linear regression model, the variable-dispersion beta regression model and the fractional logit regression model all perform well across the simulation experiments under consideration. When employing beta regression to estimate covariate effects on (0,1) response data, researchers should ensure their dispersion sub-model is properly specified, else inferential errors could arise.

## Background

In biomedical research it is common to encounter response variables which have support on the interval (0,1). These types of response variables may arise in the form of proportions/percentages, or certain types of fractions and rates. The traditional approach to analyzing these types of response data – across virtually all scientific disciplines - is via linear regression. If desired, the response variable can be transformed prior to estimation of the linear regression parameters. This transformed linear model may improve diagnostic performance; however, this may render interpretation of estimated regression parameters challenging. Alternatively, the beta distribution allows specification of a probability model for continuous random variables with support over the interval (0,1). For many years statisticians have exploited the flexibility of the beta distribution in theoretical modelling exercises; however, its use in applied research settings has not garnered equal attention. Johnson et al. [1] cite numerous instances where the beta distribution has been used in theory/practice and champion increased use of the beta distribution in applied research settings. Gupta et al. [2] also cite numerous applications where the beta distribution provides a useful probability generating model for continuous data with support on the interval (0,1). However, neither of these extensive resources on the beta distribution cites a regression modelling framework for estimating covariate effects on beta distributed response variables. Recent developments by Paulino [3], Ferrari and Cribrari-Neto [4], Smithson and Verkuilen [5] and others have resulted in a more general purpose beta regression machinery. The variable-dispersion beta regression model [5] will be used extensively in our simulation experiments, as it is particularly useful for modelling covariate effects on response variables which are assumed to follow a beta distribution. The beta regression model extends on ideas of generalized linear models [6] both in terms of their specification and estimation. Use of the beta regression model has been increasing in recent years. In slides from an unpublished presentation given by Ferrari [7], the author suggests over 100 instances where beta regression has been used in theoretical and applied research settings. Some application areas include: medicine, veterinary science, pharmacology, odontology, hydrobiology, nutritional science, forest science, waste management, education, political science, economics and finance. Clearly, embedding the beta distribution within a more general regression modelling framework has enhanced its uptake in applied research settings. A final model which we consider for estimating the average proportion/percentage/rate difference in our two-sample model is the fractional logit regression model. The fractional logit model is a popular model for fractional response variables in econometrics and was proposed (independently) by Papke and Wooldridge [8] and by Cox [9]. The fractional logit model is similar to generalized linear regression models [6]; however, it does not make any fully parametric assumptions regarding the distributional form for the response variable. Rather the fractional logit model only specifies a parametric form for the conditional mean and conditional variance of the response. The form of the conditional mean and variance functions are chosen to ensure admissible predictions/fitted-values from such models. In this case, the model specification is chosen to ensure predictions/fitted values from the fractional logit model fall in the interval (0,1). The estimator proposed by Papke and Wooldridge [8] is the one we pursue in this manuscript as they specify forms for robust variance estimators which have more desirable coverage/power properties than the more traditional quasi-likelihood models proposed by Cox [9].

Given the recent popularity of the beta regression model, especially in biomedical research, we thought it prudent to compare linear regression, beta regression, variable-dispersion beta regression and fractional logit regression models for estimating covariate effects on a response variable which lives on the interval (0,1). To accomplish this goal we conducted a Monte Carlo simulation experiment where we generated response variables following different (parametric) probability generating models. First, we considered simulating response data from the continuous beta distribution with support on (0,1). This experiment allows us to compare models when we know the beta regression model is properly specified given the response data. Specifically, we can investigate efficiency gains which may be observed from specifying an appropriate statistical model to observed response data. Additionally, we simulate response data from the discrete multinomial distribution with probability mass observed only on a finite number of points in (0,1). This experiment allows us to investigate model performance when response data is non-continuous. In this case, all models are incorrectly specified given the response data. This experiment allows us to investigate whether estimated regression models are robust to non-continuous response data. In all scenarios, we fit linear regression, beta regression, variable-dispersion beta regression, and fractional logit regression models to these randomly generated response data and compared the finite sample statistical properties of the respective estimators. We are particularly interested in the ability of each estimator to recover the average proportion/percentage/rate differences from a simple two sample design. In terms of statistical properties we will compare the respective estimators in terms of: bias, variance, type-1 error and power. Understanding the performance of these models on simulated datasets (where population parameters are known) is important for applied researchers who must discern whether to estimate covariate effects on (0,1) response data using the traditional linear regression model or more novel regression models, such as beta regression, variable-dispersion beta regression and fractional logit regression models.

## Methods

### Statistical methods

#### ***The linear regression model***

The linear regression model is a workhorse of applied statisticians. It is used to model the effect of continuous/categorical covariates on a scalar response (assumed to be generated according to a Gaussian probability model). Thorough introductions to the linear regression model are given in Weisberg [10], McCullagh and Nelder [6], and White [11].

In this study we consider a simple two sample problem, re-cast under a regression framework, such that our response variable is modelled as a function of a single intercept parameter and a single slope parameter. The linear model and its conditional mean function look as follows:

$$Y_{i}=\beta_{0}+\beta_{1}X_{i1}+\epsilon_{i}$$

$$E\left(\left(Y_{i}\right|X_{i}\right)=\beta_{0}+\beta_{1}X_{i1}$$

The notation above suggests that we observe a vector of response variables, Y_1_…Y_n_. Further, we have information on a single binary covariate, X_i_ ∈ {0,1}, again for i = 1…n. The regression coefficients β_0_ and β_1_ are estimated from the data. Estimation and inferential procedures are justified given that the following assumptions are satisfied [11]:

1. The model is properly specified

2. X is a non-stochastic and finite dimensional (n by p) matrix with n ≥ p

3. (X^T^X) is non-singular and hence invertible

4. E(ϵ_i_) = 0 ∀ (i = 1…n)

5. ϵ_i_ ~ Normal(0, σ^2^) ∀ (i = 1…n)

In our experiment, we are interested in the ability of the linear regression estimator to recover the average proportion/percentage/rate difference given our simple two sample design. Taking linear combinations of the estimated model parameters we arrive at the following estimator:

$$\Delta =E\left(Y_{i}\;|\;X_{i1}=1\right)-E\left(Y_{i}\;|\;X_{i1}=0\right)=\beta_{1}$$

Therefore a test of ∆ = 0 is equivalent to a test of β_1_ = 0. In this simulation we carry out such a test using a Wald statistic, W, which follows an asymptotic standard normal distribution. We reject the null hypothesis in instances where |W| > 1.96 (corresponding to an α = 5% significance threshold).

#### ***The beta regression model (and some extensions)***

The beta regression model was proposed by Paulino [3], Ferrari and Cribrari-Neto [4], and Smithson and Verkuilen [5] for modelling covariate effects on a continuous response variable which assumes support on the interval (0,1).

The beta distribution is thoroughly described in Johnson et al. [1] and Gupta [2]. The beta density is a very flexible density, assuming support on the interval (0,1). The most common parameterization of the beta density is in terms of its two shape parameters {p,q}:

$$f\left(y;p,q\right)=\frac{\Gamma \left(p+q\right)}{\Gamma \left(p\right)\Gamma \left(q\right)}y^{p-1}{\left(1-y\right)}^{q-1}$$

In the parameterization given above we assume p > 0, q > 0, y ∈ (0,1) and use (·) to denote the gamma function (a generalization of the factorial function to non-integer arguments). The density assumes probability mass on the interval (0,1) and is zero elsewhere. Further, under this parameterization we define the mean and variance of the random variable, Y, as follows:

$$E\left(Y\right)=\frac{p}{p+q}$$

$$\text{VAR}\left(Y\right)=\frac{\mathrm{pq}}{{\left(p+q\right)}^{2}\left(p+q+1\right)}$$

Above E(·) and VAR(·) denote the expectation and variance operators, with respect to the given beta distribution. In regression modelling, it is more common to parameterize the density in terms of a mean (μ) and dispersion parameter (*ϕ*) instead of two shape parameters, {p,q}. In this parameterization we have the following relationships:

$$\mu =\frac{p}{p+q}$$

ϕ=p+q

This implies: *p* = *μϕ* and *q* = (1 – *μ*)*ϕ*.

Given the above relationships we can derive the mean and the variance of the beta density in terms of a mean and dispersion parameter as follows:

$$E\left(Y\right)=\mu$$

$$\text{VAR}\left(Y\right)=\frac{V\left(\mu \right)}{1+\phi }=\frac{\mu \left(1-\mu \right)}{1+\phi }$$

Given a fixed value for the mean, the larger the value of *ϕ* the smaller the variance of the response variable, Y (and vice-versa). Under this new parameterization, in terms of a mean and dispersion parameter, the density of Y looks as follows:

$$f\left(y;\mu ,\phi \right)=\frac{\Gamma \left(\phi \right)}{\Gamma \left(\mu ,\phi \right)\Gamma \left(\left(1-\mu \right)\phi \right)}y^{\mu \phi -1}{\left(1-y\right)}^{\left(1-\mu \right)\phi -1}$$

**Figure 1:**
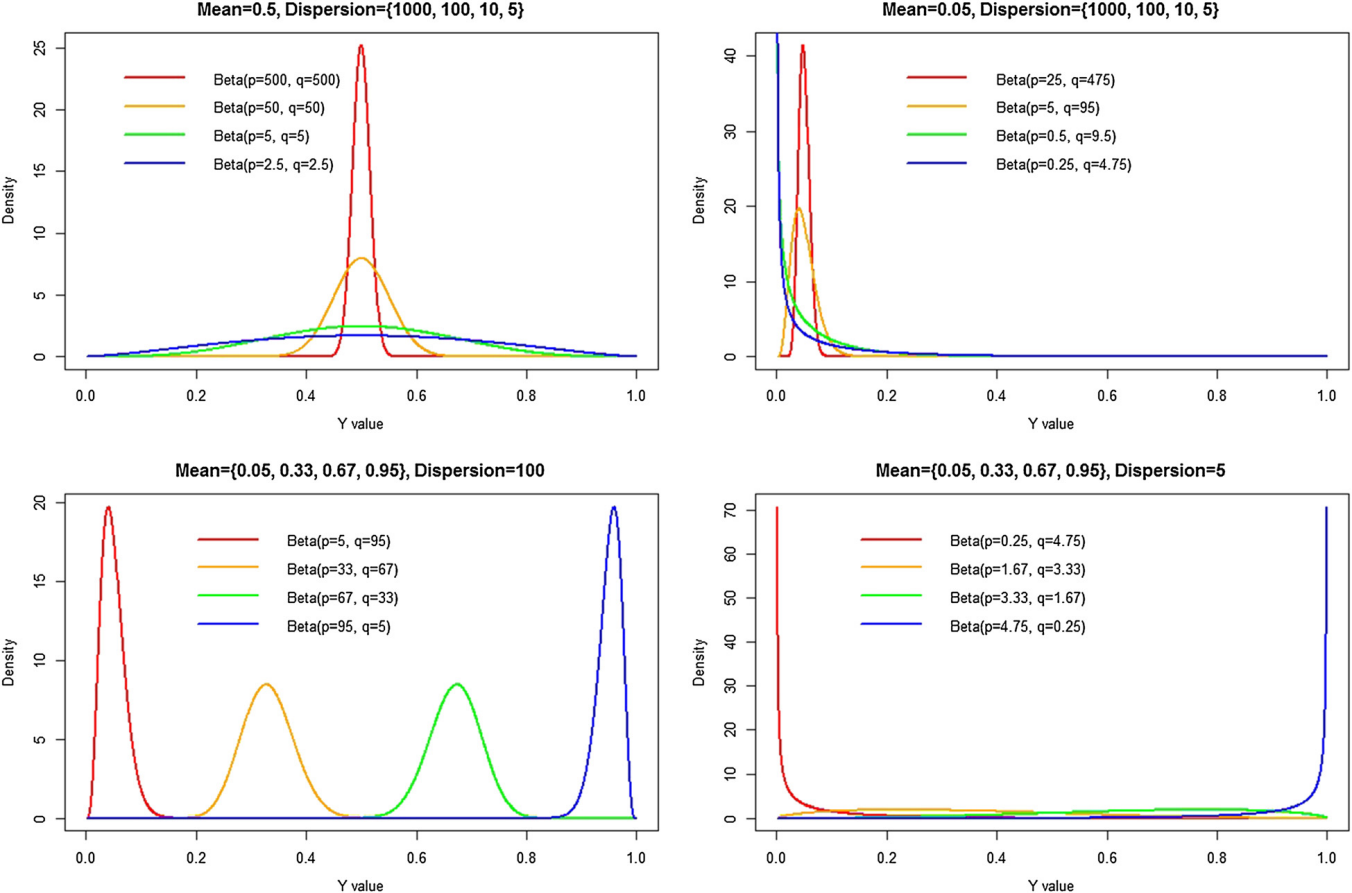
**Various forms of the beta density for varying shape parameters {p,q}.** Top left panel: We fix the mean equal to 0.5 and plot the resulting beta densities for varying dispersion parameters. Top right panel: We fix the mean equal to 0.05 and plot the resulting beta densities for varying dispersion parameters. Bottom left panel: We fix the dispersion parameter equal to 100 and plot the resulting beta densities for varying mean parameters. Bottom right panel: We fix the dispersion parameter equal to 5 and plot the resulting beta densities for varying mean parameters.

In Figure 1, we graphically represent some of the forms the beta density can take on for different values of {p,q}, or alternatively, {*μ*, *ϕ*}.

The beta regression model, and the variable-dispersion extensions which we will discuss in this study are being increasingly utilized to model covariate effects on response variables observed on the interval (0,1). The beta regression model is an obvious choice for modelling response data which follow a beta distribution. Consider the scenario where we observe response data Y_1…_Y_n_ on the interval (0,1). The beta regression model assumes that the mean of these random variables, can be represented in the following form:

$$g\left(\mu_{i}\right)=\eta_{i}=\beta_{0}+\beta_{1}X_{i1}$$

Our link function g(·) can be any function which is strictly monotone, twice differentiable, and maps the response variable observed on the interval (0,1) to the real line. The most commonly used link function in beta regression is the logit link. Alternative link functions include: the probit, the complementary log-log, the log-log and the Cauchy link. In general, any inverse cumulative distribution function will be an appropriate link function in a beta regression framework as they act to map the interval (0,1) to the real line.

The components of the basic beta regression model can be summarized as:

1. A response variable from a beta distribution

2. A linear predictor, η_i_

3. A suitable link function, such that: *E*(*Y*_*i*_|*X*_*i*_) = *g*(*μ*_i_) = *η*_i_

Given the above components, the log-likelihood of the beta regression model can be written as follows:

$$\begin{array}{ll} \;\mathrm{LL}\left(\mu_{i},\phi \right)= & \text{log}\Gamma \left(\phi \right)-\text{log}\Gamma \left(\mu_{i}\phi \right)-\text{log}\left(\left(1-\mu_{i}\right)\phi \right)\\ & +\left(\mu_{i}\phi -1\right)\text{log}\left(y_{i}\right)\\ & +\left\{\left(1-\mu_{i}\right)\phi -1\right\}\text{log}\left(1-y_{i}\right) \end{array}$$

The log-likelihood function can be maximized numerically as described in Ferrari and Cribrari-Neto [4]. The mean and dispersion parameter estimates are known to be biased, especially in small samples. Kosmidis and Firth [12] discuss the issue of finite sample bias in beta regression. The authors propose a general purpose algorithm for producing bias-reduced and bias-corrected parameter estimates via adjustments to the score function. In our simulation experiment we estimate parameters from the beta regression model via standard maximum likelihood (ML) methods, as well as the bias-reduced (BR) and bias-corrected (BC) methods. In our simulation experiments we employ the simple ML estimators; however, we note that BC/BR methods may improve type-1 error rates in small sample situations.

The beta regression model proposed above assumes that the dispersion parameter is constant for all individuals under consideration. In many biomedical applications this may be an unrealistic assumption (especially if one expects a non-zero mean difference across categorical groups). As its name implies, the variable-dispersion beta regression model [5] allows the value of the dispersion parameter to vary across individuals. Further, the value of the dispersion parameter can actually be modelled as a function of covariates. The variable-dispersion beta regression model is a type of double-index regression model [13], as it contains two regression equations, one modelling the mean as a function of covariates and the other modelling the dispersion as a function of covariates.

Again, we consider the scenario where we observe response data Y_1…_Y_n_ on the interval (0,1). The variable-dispersion beta regression model assumes that the mean and dispersion of these random variables can be represented in the following form:

$$g\left(\mu_{i}\right)=\eta_{i}=\beta_{0}+\beta_{1}X_{i1}$$

$$h\left(\phi_{i}\right)=\zeta_{i}=\gamma_{0}+\gamma_{1}X_{i1}$$

Once again, we assume that both g(·) and h(·) are strictly monotonic, twice differentiable functions which act to map the mean, μ_i_, and the dispersion, *ϕ*_*i*_, to the real line. Once again, suitable choices of g(·) include the following link functions: logit, probit, complementary log-log, log-log, Cauchy or any other inverse cumulative distribution function. The link function for h(·) is typically chosen to be the log link. The identity link can also be used; however, it has the undesirable property of possibly suggesting non-positive values of *ϕ*_*i*_.

The log-likelihood function for the variable-dispersion beta regression model can be numerically maximized and is subject to similar finite sample biases as the basic beta regression model. Below, we illustrate the log-likelihood function for this model:

$$\begin{array}{ll} \;\mathrm{LL}\left(\mu_{i},\phi \right)= & \text{log}\Gamma \left(\phi_{i}\right)-\text{log}\Gamma \left(\mu_{i}\phi_{i}\right)-\text{log}\left(\left(1-\mu_{i}\right)\phi_{i}\right)\\ & +\left(\mu_{i}\phi_{i}-1\right)\text{log}\left(y_{i}\right)\\ & +\left\{\left(1-\mu_{i}\right)\phi_{i}-1\right\}\text{log}\left(1-y_{i}\right) \end{array}$$

In the case of both the beta regression model and the variable-dispersion (double-index) beta regression models, estimates of mean and dispersion parameters {β,γ} are achieved by numerically solving the likelihood equations given above. The resulting parameter estimates are asymptotically normally distributed and take the following form:

β^γ^~MVNβγ,C-1

Further,

$$C^{-1}=C^{-1}\left(\beta ,\gamma \right)=\left(\begin{array}{cc} C^{\beta \beta } & C^{\beta \gamma }\\ C^{\gamma \beta } & C^{\gamma \gamma } \end{array}\right)$$

For our purposes it suffices to realize that the estimators of the mean and dispersion parameters are consistent estimators of their target parameters and are distributed according to a multivariate normal distribution, with variance-covariance matrix C^-1^. Detailed derivations of these formulas (particularly pertaining to the forms of the C^-1^ matrix) are given in Ferrari and Cribrari-Neto [4].

Again, we are interested in the ability of the (variable-dispersion) beta regression estimator to recover the average proportion/percentage/rate difference given our two sample design. In all of our simulation experiments we assume a logit link for the mean function. The (default) identity link is used in the beta regression modelling context and the log link is used in the variable-dispersion beta regression context. In all scenarios, our target of inference is the average proportion/percentage/rate difference and we view the terms in the dispersion sub-model as a nuisance. A point estimator of the proportion/percentage/rate difference from the beta regression model is:

$$\Delta =\text{log}\left(\frac{1}{1+\text{exp}\left(-\beta_{0}-\beta_{1}X_{i1}\right)}\right)-\text{log}\left(\frac{1}{1+\text{exp}\left(-\beta_{0}\right)}\right)$$

We use the delta method to estimate the variance and standard error of this estimator, respectively. We construct a Wald style test of the null hypothesis that ∆ = 0. The Wald statistic, W, is computed as the ratio of the difference in proportion/percentage/rates over the estimated standard error. The test statistic is presumed to follow an asymptotic standard normal distribution. Again, we use a 5% critical threshold for rejecting the null hypothesis (this corresponds to rejection of H_0_ if |W| > 1.96)

#### ***The fractional logit regression model***

The final methodology we consider for estimating average proportion/percentage/rate differences in our two-sample design is the fractional logit regression model [8,9]. The fractional logit regression model is most commonly encountered in the econometrics literature and has been demonstrated as being an effective means for estimating covariate effects on a response variable which lives on (0,1). Hence we consider it in this manuscript – as a result, introducing health services researchers to yet another plausible strategy for modelling proportions/percentages/fractions/rates.

The fractional logit regression model is considered a quasi-parametric regression model. In other words, the fractional logit regression model does not make any parametric assumption regarding the distribution of the response variable being modelled; rather, it makes assumptions regarding only the first two conditional moments of the response variable – the conditional mean and the conditional variance. The choice of the conditional mean and conditional variance function are typically made to ensure that predictions/fitted-values from the specified model are admissible. In our case, this implies that the predictions/fitted-values fall in the interval (0,1).

As mentioned above, quasi-likelihood models typically only make assumptions regarding the first two conditional moments of the response variable [6, 9]. The conditional variance is assumed to be a known function of the mean (up to a scale parameter) and the conditional mean function therein is assumed to be a function of unknown model parameters:

$$V\left(\left(Y_{i}\right|X_{i}\right)=\sigma^{2}\nu \left(\mu_{i}\right)$$

$$E\left(\left(Y_{i}\right|X_{i}\right)=g\left(\mu_{i}\right)=\beta_{0}+\beta_{1}X_{i1}+\ldots +\beta_{p}X_{\mathrm{ip}}$$

In the first Equation V(·) denotes the variance operator, σ^2^ is a scale parameter which is estimated from observed data. ν(·) is a known variance function, and μ_i_ is the mean function. In the second Equation E(·) denotes the variance operator, g(·) is a known link function and β_j_ represent the unknown mean function parameters which must be estimated from the data. Y_i_ and X_i_ represent the response variable and observed covariates, respectively.

In describing the fractional logit model we adopt the terminology of Papke and Wooldridge [8]. Our chief assumption relates to the specification of the conditional mean function, namely:

$$E\left(Y_{i}\right)=h\left(\mu_{i}\right)$$

Generally, h(·) is a known function which maps our real valued linear predictor into the interval (0,1). Again, their exist many plausible function which could accomplish this goal, in this manuscript we choose h(·) to be the logistic function and arrive at the fractional logit model. That is:

$$h\left(\mu_{i}\right)=\frac{\text{exp}\left(\mu_{i}\right)}{1+\text{exp}\left(\mu_{i}\right)}=\frac{1}{1+\text{exp}\left(-\mu_{i}\right)}$$

Further, the conditional variance of the response variable is assumed to be:

$$V\left(\left(Y_{i}\right|x_{i}\right)=\sigma^{2}\left(h\left(\mu_{i}\right)\left(1-h\left(\mu_{i}\right)\right)\right)$$

Papke and Wooldridge [8] argue that this conditional variance assumption is too restrictive for modelling response data with support over (0,1). Therefore, in their manuscript they offer two alternative strategies: first, using robust/sandwich estimators of the variance-covariance matrix and second, adjusting the estimated variance-covariance matrix by the Pearson scale adjustment factor. We considered both approaches; however, noted little difference in performance between the two estimators of the variance-covariance matrix. Hence, we report on only the fractional logit model with sandwich/robust variance-covariance matrix.

Parameter estimation under the fractional logit model proceeds by maximizing the following Bernoulli quasi-likelihood function:

$$\mathrm{LL}=y_{i}*\text{log}\left(h\left(\mu_{i}\right)\right)+\left(1-y_{i}\right)*\text{log}\left(h\left(\mu_{i}\right)\right)$$

#### ***Monte Carlo simulation design***

The goal of this simulation experiment is to compare the properties of the linear regression model, the beta regression model, the variable-dispersion beta regression model and the fractional logit regression model at recovering estimates of average proportion/percentage/rate differences from a simple two sample design. In all experiments we simulate data from parametric probability generating models such that the observed response data is on the interval (0,1). Subsequently, we estimate covariate effects on the response variable using one of four regression models: the linear regression model, the beta regression model, the (double-index) variable-dispersion beta regression model and the fractional logit regression model. Given estimates of average proportion/percentage/rate differences from the respective models, we compare statistical properties of the respective estimators, such as: bias, variance, type-1 error and power [14,15]. We investigate finite sample performance of each of the estimators by varying the sample size within each unique simulation experiment. In all scenarios, the sample size in group 1 is set equal to the sample size in group 2. Group specific sample sizes under consideration in this simulation are: 25, 100, 250, and 750. The total sample size for a given simulation experiment is double the group-specific sample size (as this experiment assumes a 2-sample design). In each instance we consider 20,000 replications of each experiment. We choose 20,000 replicate simulations such that coverage in the type-1 error experiments is based off of approximately 1000 rejections of a true null hypothesis. We present mean estimates of bias, variance, type-1 error and power averaged across the 20,000 replicate simulations. Further we present Monte Carlo error estimates of bias, variance and power. Detailed derivations of Monte Carlo error are described in White [16]. The “seeds” which govern the pseudo-randomness of the various Monte Carlo experiments are given in the attached R/SAS codes.

The first parametric probability model which we consider for generating response data on the interval (0,1) is the beta distribution. Table 1 describes the parameter values used to generate randomly simulated beta response variables. The response variables are generated such that certain mean and dispersion properties are achieved. For example, mean differences of zero are used to assess the type-1 error rates of respective estimators (for both fixed and varying dispersion). Further, non-zero mean differences are used to assess power (again for both fixed and varying dispersion). In this experiment response data are generated as independent draws from the respective beta distributions. That is, observations within and between the two samples are independently distributed. Within the type-1 error and power experiment frameworks, respectively, we have 3 sub-experiments: the first set of experiments consider the scenario where the central tendency of the simulated response distribution is near the center of the support (0.5); the second set of experiments considers the effect of shifting the central tendency to the right such that it is centered near 0.25; and finally, the last experiment considers the effect of shifting the central tendency to the boundary of the support, near 0.05. As sub-scenarios we vary the shape of the beta distribution when data are simulated from the center, right-center and far-right of the support, considering scenarios where the simulated data are symmetric and other scenarios where the simulated data is highly skewed. As the data are beta distributed we expect the beta regression models to perform well in all scenarios; however, we anticipate that the linear model will perform well when data are symmetric and unimodal. That is, we expect the linear model to perform well as the shape/rate parameters both become large and as the ratio of the shape/rate parameters approach 1 (resulting in a symmetric and unimodal beta distribution – which converges to that of a normal distribution).

**Table 1:** Description of 24 simulation experiments where the response variable is distributed according a beta distribution with the following mean and dispersion parameters in each respective group (or alternatively parameterized in terms of its two shape parameters – p and q – in each group)

	**μ**_**0**_	***φ***_**0**_	**μ**_**1**_	***φ***_**1**_	**p**_**0**_	**q**_**0**_	**p**_**1**_	**q**_**1**_
Type-1 error experiments	0.50	5	0.50	5	2.5	2.5	2.5	2.5
	0.50	5	0.50	10	2.5	2.5	5	5
	0.50	100	0.50	100	50	50	50	50
	0.50	100	0.50	200	50	50	100	100
	0.25	5	0.25	5	1.25	3.75	1.25	3.75
	0.25	5	0.25	10	1.25	3.75	2.50	7.50
	0.25	100	0.25	100	25	75	25	75
	0.25	100	0.25	200	25	75	50	150
	0.05	5	0.05	5	0.25	4.75	0.25	4.75
	0.05	5	0.05	10	0.25	4.75	0.50	9.50
	0.05	100	0.05	100	5	95	5	95
	0.05	100	0.05	200	5	95	10	190
Power experiments	0.5	5	0.525	5	2.5	2.5	2.625	2.375
	0.5	5	0.525	10	2.5	2.5	5.25	4.75
	0.5	100	0.525	100	50	50	52.5	47.5
	0.5	100	0.525	200	50	50	105	95
	0.25	5	0.275	5	1.25	3.75	1.375	3.625
	0.25	5	0.275	10	1.25	3.75	2.75	7.25
	0.25	100	0.275	100	25	75	27.5	62.5
	0.25	100	0.275	200	25	75	55	145
	0.05	5	0.075	5	0.25	4.75	0.375	4.625
	0.05	5	0.075	10	0.25	4.75	0.75	9.25
	0.05	100	0.075	100	5	95	7.5	92.5
	0.05	100	0.075	200	5	95	15	185

The next parametric probability model under consideration is the discrete multinomial model which takes probability mass only on a finite number of points on the interval (0,1). More specifically, we assume our response variable Y_i_ can take on the following values:

$$Y_{i}\in \left\{0.05,0.15,0.25,0.35,0.45,0.55,0.65,0.75,0.85,0.95\right\}$$

That said, we do not assume the probability of assuming these values is necessarily uniform. Rather, we assign a vector of probabilities to these points, corresponding to the relative likelihood that the response variable assumes that particular value. Table 2 describes the particular probability vectors used to generate response variables for each group in our two sample design. Once again, we vary the expected value of the response to assess differences in type-1 error rates and power across our linear regression, beta regression, (double-index) variable-dispersion beta regression and fractional logit regression models. Again, in this experiment response data are generated as independent draws from the respective multinomial distributions. That is, observations within and between the two samples are independently distributed.

**Table 2:** Description of 4 simulation experiments where the response variable is distributed according to a discrete multinomial distribution on the points {0.05, 0.15, 0.25, 0.35, 0.45, 0.55, 0.65, 0.75, 0.85, 0.95} with corresponding probabilities of occurrence listed in the table for each of the two groups under consideration

	**Group 0 – multinomial response probabilities**										**Group 1 – multinomial response probabilities**									
Y_i_ values	0.05	0.15	0.25	0.35	0.45	0.55	0.65	0.75	0.85	0.95	0.05	0.15	0.25	0.35	0.45	0.55	0.65	0.75	0.85	0.95
Type-1 error experiments	0.00	0.025	0.025	0.15	0.30	0.30	0.15	0.025	0.025	0.00	0.00	0.025	0.025	0.15	0.30	0.30	0.15	0.025	0.025	0.00
	0.40	0.20	0.10	0.10	0.10	0.05	0.05	0.00	0.00	0.00	0.40	0.20	0.10	0.10	0.10	0.05	0.05	0.00	0.00	0.00
Power experiments	0.00	0.025	0.025	0.15	0.30	0.30	0.15	0.025	0.025	0.00	0.00	0.00	0.025	0.025	0.15	0.30	0.30	0.15	0.025	0.025
	0.40	0.20	0.10	0.10	0.10	0.05	0.05	0.00	0.00	0.00	0.00	0.40	0.20	0.10	0.10	0.10	0.05	0.05	0.00	0.00

#### ***Statistical software***

This simulation experiment was conducted using R version 3.02 [17] and results were also verified using SAS 9.3 [18].

Simulation of the beta and multinomial response variables were carried out using the rbeta() and rmultinom() functions, respectively. Linear regression modelling was performed using the lm() function. Beta regression was performed using the betareg() function in the betareg library [13]. Fractional logit regression models were estimated using the glm() function and the sandwich() function [19]. Standard errors for the proportion/percentage/rate differences from beta regression and fractional logit regression models were calculated using the deltamethod() function in the msm library [20].

SAS PROC NLMIXED was used to specify the linear regression model, beta regression model, variable-dispersion beta-regression model and fraction logit regression model likelihood equations, respectively, and model parameters were estimated via likelihood methods.

All R and SAS code used to conduct this simulation can be obtained by contacting the corresponding author.

## Results

Detailed results of the Monte Carlo simulation study are given in Tables 3, 4, 5 and 6. Tables 3 and 4 describe the type-1 error and power experiments, respectively, given response data simulated according to independent draws from various parameterizations of the beta distribution. Tables 5 and 6 describe the type-1 error and power experiments, respectively, given response data simulated according to independent draws from various parameterizations of the multinomial distribution.

**Table 3:** Mean estimates from 20000 replicate simulations of bias (MC error of bias), variance (MC error of variance) and type-1 error (MC error type-1 error), from the fitted linear, beta, variable-dispersion beta and fractional logit regression models estimated on the beta distributed response data (Type 1 error experiments)

					**Linear regression model**						**Beta regression model**						**Variable dispersion beta regression model**						**Fractional logit regression model**					
**N0 = N1**	**μ**_**0**_	***φ***_**0**_	**μ**_**1**_	***φ***_**1**_	**Bias**	**MC error bias**	**Variance**	**MC error variance**	**Type-1 Error**	**MC Error Type-1 Error**	**Bias**	**MC error bias**	**Variance**	**MC error variance**	**Type-1 Error**	**MC Error Type-1 Error**	**Bias**	**MC error bias**	**Variance**	**MC error variance**	**Type-1 Error**	**MC Error Type-1 Error**	**Bias**	**MC error bias**	**Variance**	**MC error variance**	**Type-1 Error**	**MC Error Type-1 Error**
25	0.5	5	0.5	5	-4.18E-04	4.10E-04	3.34E-03	3.35E-05	0.050	0.002	-4.52E-04	4.06E-04	3.11E-03	2.99E-05	0.063	0.002	-4.57E-04	4.07E-04	3.09E-03	2.97E-05	0.064	0.002	-4.18E-04	4.10E-04	3.20E-03	3.29E-05	0.061	0.002
100	0.5	5	0.5	5	2.07E-04	2.05E-04	8.33E-04	8.15E-06	0.050	0.002	1.65E-04	2.03E-04	8.06E-04	7.41E-06	0.052	0.002	1.61E-04	2.03E-04	8.05E-04	7.40E-06	0.053	0.002	2.07E-04	2.05E-04	8.25E-04	8.11E-06	0.053	0.002
250	0.5	5	0.5	5	-1.72E-04	1.29E-04	3.33E-04	3.24E-06	0.050	0.002	-1.57E-04	1.27E-04	3.25E-04	2.95E-06	0.052	0.002	-1.61E-04	1.28E-04	3.24E-04	2.94E-06	0.052	0.002	-1.72E-04	1.29E-04	3.32E-04	3.23E-06	0.051	0.002
750	0.5	5	0.5	5	2.88E-05	7.45E-05	1.11E-04	1.08E-06	0.050	0.002	1.20E-05	7.35E-05	1.09E-04	9.87E-07	0.049	0.002	1.15E-05	7.35E-05	1.09E-04	9.87E-07	0.049	0.002	2.88E-05	7.45E-05	1.11E-04	1.08E-06	0.050	0.002
25	0.5	100	0.5	100	3.16E-05	9.98E-05	1.98E-04	1.00E-05	0.051	0.002	3.25E-05	9.98E-05	1.90E-04	9.81E-06	0.062	0.002	3.24E-05	9.98E-05	1.90E-04	9.80E-06	0.062	0.002	3.16E-05	9.98E-05	1.91E-04	9.82E-06	0.061	0.002
100	0.5	100	0.5	100	-1.44E-05	4.99E-05	4.95E-05	2.44E-06	0.051	0.002	-1.38E-05	4.99E-05	4.90E-05	2.43E-06	0.054	0.002	-1.38E-05	4.99E-05	4.90E-05	2.43E-06	0.054	0.002	-1.44E-05	4.99E-05	4.90E-05	2.43E-06	0.053	0.002
250	0.5	100	0.5	100	6.80E-06	3.13E-05	1.98E-05	1.01E-06	0.049	0.002	6.67E-06	3.12E-05	1.97E-05	1.01E-06	0.049	0.002	6.67E-06	3.12E-05	1.97E-05	1.01E-06	0.049	0.002	6.80E-06	3.13E-05	1.97E-05	1.01E-06	0.049	0.002
750	0.5	100	0.5	100	-1.67E-06	1.82E-05	6.66E-06	6.47E-07	0.052	0.002	-1.62E-06	1.82E-05	6.65E-06	6.55E-07	0.053	0.002	-1.62E-06	1.82E-05	6.65E-06	6.55E-07	0.053	0.002	-1.67E-06	1.82E-05	6.65E-06	6.54E-07	0.053	0.002
25	0.25	5	0.25	5	-2.08E-04	3.52E-04	2.50E-03	3.74E-05	0.049	0.002	-7.51E-05	3.27E-04	2.05E-03	3.10E-05	0.062	0.002	-1.59E-04	3.48E-04	2.33E-03	3.25E-05	0.060	0.002	-2.08E-04	3.52E-04	2.40E-03	3.67E-05	0.060	0.002
100	0.25	5	0.25	5	2.77E-04	1.77E-04	6.26E-04	9.09E-06	0.050	0.002	2.83E-04	1.64E-04	5.28E-04	7.71E-06	0.053	0.002	3.26E-04	1.75E-04	6.06E-04	8.12E-06	0.053	0.002	2.77E-04	1.77E-04	6.20E-04	9.04E-06	0.053	0.002
250	0.25	5	0.25	5	1.11E-04	1.12E-04	2.50E-04	3.64E-06	0.049	0.002	1.38E-04	1.03E-04	2.12E-04	3.10E-06	0.051	0.002	1.14E-04	1.10E-04	2.44E-04	3.27E-06	0.050	0.002	1.11E-04	1.12E-04	2.49E-04	3.63E-06	0.050	0.002
750	0.25	5	0.25	5	4.20E-05	6.44E-05	8.33E-05	1.22E-06	0.048	0.002	2.86E-05	5.94E-05	7.09E-05	1.05E-06	0.050	0.002	4.40E-05	6.38E-05	8.15E-05	1.10E-06	0.049	0.002	4.20E-05	6.44E-05	8.32E-05	1.22E-06	0.048	0.002
25	0.25	100	0.25	100	2.34E-05	8.69E-05	1.48E-04	8.84E-06	0.050	0.002	1.31E-05	8.66E-05	1.41E-04	8.50E-06	0.060	0.002	2.39E-05	8.69E-05	1.42E-04	8.58E-06	0.060	0.002	2.34E-05	8.69E-05	1.42E-04	8.65E-06	0.060	0.002
100	0.25	100	0.25	100	9.32E-06	4.31E-05	3.71E-05	2.18E-06	0.051	0.002	1.03E-05	4.30E-05	3.65E-05	2.13E-06	0.053	0.002	9.62E-06	4.31E-05	3.67E-05	2.15E-06	0.053	0.002	9.32E-06	4.31E-05	3.67E-05	2.17E-06	0.053	0.002
250	0.25	100	0.25	100	-7.06E-06	2.73E-05	1.49E-05	9.05E-07	0.049	0.002	-8.38E-06	2.72E-05	1.47E-05	8.93E-07	0.050	0.002	-7.25E-06	2.73E-05	1.48E-05	9.01E-07	0.049	0.002	-7.06E-06	2.73E-05	1.48E-05	9.05E-07	0.050	0.002
750	0.25	100	0.25	100	1.44E-05	1.58E-05	5.00E-06	1.36E-07	0.049	0.002	1.30E-05	1.58E-05	4.99E-06	1.63E-07	0.051	0.002	1.44E-05	1.58E-05	4.99E-06	1.37E-07	0.050	0.002	1.44E-05	1.58E-05	4.99E-06	1.39E-07	0.049	0.002
25	0.05	5	0.05	5	3.45E-04	1.78E-04	6.35E-04	4.42E-05	0.042	0.001	1.05E-04	9.94E-05	1.96E-04	2.44E-05	0.040	0.001	3.40E-04	1.73E-04	6.24E-04	3.82E-05	0.036	0.001	3.45E-04	1.78E-04	6.09E-04	4.33E-05	0.054	0.002
100	0.05	5	0.05	5	-7.35E-05	8.93E-05	1.59E-04	1.10E-05	0.051	0.002	-3.62E-05	4.83E-05	4.68E-05	5.98E-06	0.048	0.002	-6.70E-05	8.80E-05	1.56E-04	9.75E-06	0.048	0.002	-7.35E-05	8.93E-05	1.57E-04	1.09E-05	0.053	0.002
250	0.05	5	0.05	5	2.00E-05	5.58E-05	6.32E-05	4.40E-06	0.049	0.002	4.10E-05	3.03E-05	1.84E-05	2.38E-06	0.050	0.002	1.63E-05	5.53E-05	6.21E-05	3.92E-06	0.048	0.002	2.00E-05	5.58E-05	6.29E-05	4.39E-06	0.050	0.002
750	0.05	5	0.05	5	6.80E-06	3.25E-05	2.11E-05	1.49E-06	0.050	0.002	-6.04E-06	1.75E-05	6.13E-06	8.98E-07	0.050	0.002	6.78E-06	3.21E-05	2.07E-05	1.34E-06	0.050	0.002	6.80E-06	3.25E-05	2.11E-05	1.49E-06	0.050	0.002
25	0.05	100	0.05	100	-4.70E-05	4.37E-05	3.76E-05	5.33E-06	0.050	0.002	-5.81E-05	4.19E-05	3.32E-05	4.40E-06	0.062	0.002	-4.70E-05	4.36E-05	3.64E-05	4.92E-06	0.059	0.002	-4.70E-05	4.37E-05	3.61E-05	5.22E-06	0.060	0.002
100	0.05	100	0.05	100	2.39E-05	2.16E-05	9.41E-06	1.36E-06	0.049	0.002	2.17E-05	2.07E-05	8.54E-06	1.14E-06	0.052	0.002	2.38E-05	2.16E-05	9.32E-06	1.26E-06	0.050	0.002	2.39E-05	2.16E-05	9.31E-06	1.35E-06	0.052	0.002
250	0.05	100	0.05	100	-3.24E-05	1.37E-05	3.82E-06	7.23E-07	0.050	0.002	-3.21E-05	1.31E-05	3.38E-06	9.34E-07	0.050	0.002	-3.24E-05	1.37E-05	3.83E-06	6.98E-07	0.051	0.002	-3.24E-05	1.37E-05	3.81E-06	7.43E-07	0.051	0.002
750	0.05	100	0.05	100	-2.60E-06	7.93E-06	1.00E-06	0.00E + 00	0.052	0.002	-3.87E-06	7.60E-06	1.00E-06	0.00E + 00	0.052	0.002	-2.63E-06	7.93E-06	1.00E-06	0.00E + 00	0.052	0.002	-2.60E-06	7.93E-06	1.00E-06	0.00E + 00	0.052	0.002
25	0.5	5	0.5	10	-2.22E-04	3.59E-04	2.57E-03	3.12E-05	0.052	0.002	-2.37E-04	3.62E-04	2.46E-03	3.01E-05	0.065	0.002	-2.24E-04	3.58E-04	2.40E-03	2.81E-05	0.064	0.002	-2.22E-04	3.59E-04	2.47E-03	3.06E-05	0.062	0.002
100	0.5	5	0.5	10	-1.92E-04	1.80E-04	6.44E-04	7.71E-06	0.051	0.002	-2.03E-04	1.82E-04	6.40E-04	7.59E-06	0.055	0.002	-2.09E-04	1.79E-04	6.26E-04	7.12E-06	0.054	0.002	-1.92E-04	1.80E-04	6.38E-04	7.68E-06	0.054	0.002
250	0.5	5	0.5	10	-9.90E-05	1.13E-04	2.57E-04	3.04E-06	0.050	0.002	-7.63E-05	1.14E-04	2.58E-04	3.00E-06	0.054	0.002	-6.83E-05	1.12E-04	2.52E-04	2.81E-06	0.052	0.002	-9.90E-05	1.13E-04	2.56E-04	3.03E-06	0.051	0.002
750	0.5	5	0.5	10	2.83E-05	6.59E-05	8.58E-05	1.02E-06	0.052	0.002	4.12E-05	6.65E-05	8.62E-05	1.01E-06	0.053	0.002	3.14E-05	6.54E-05	8.43E-05	9.47E-07	0.053	0.002	2.83E-05	6.59E-05	8.57E-05	1.02E-06	0.053	0.002
25	0.5	100	0.5	200	-2.30E-05	8.62E-05	1.49E-04	9.17E-06	0.051	0.002	-2.33E-05	8.63E-05	1.43E-04	9.01E-06	0.061	0.002	-2.36E-05	8.62E-05	1.43E-04	8.97E-06	0.061	0.002	-2.30E-05	8.62E-05	1.43E-04	8.99E-06	0.061	0.002
100	0.5	100	0.5	200	9.13E-05	4.29E-05	3.72E-05	2.25E-06	0.046	0.001	9.09E-05	4.29E-05	3.68E-05	2.25E-06	0.048	0.002	9.09E-05	4.29E-05	3.68E-05	2.24E-06	0.048	0.002	9.13E-05	4.29E-05	3.68E-05	2.24E-06	0.048	0.002
250	0.5	100	0.5	200	-4.36E-05	2.74E-05	1.49E-05	9.42E-07	0.051	0.002	-4.35E-05	2.74E-05	1.48E-05	9.43E-07	0.052	0.002	-4.34E-05	2.74E-05	1.48E-05	9.39E-07	0.052	0.002	-4.36E-05	2.74E-05	1.48E-05	9.41E-07	0.052	0.002
750	0.5	100	0.5	200	1.09E-06	1.58E-05	5.00E-06	1.47E-07	0.052	0.002	8.86E-07	1.58E-05	5.00E-06	1.48E-07	0.052	0.002	8.93E-07	1.58E-05	5.00E-06	1.49E-07	0.052	0.002	1.09E-06	1.58E-05	5.00E-06	1.49E-07	0.052	0.002
25	0.25	5	0.25	10	-3.68E-04	3.11E-04	1.93E-03	3.36E-05	0.050	0.002	2.27E-02	2.96E-04	1.67E-03	2.86E-05	0.102	0.002	-1.82E-05	3.09E-04	1.81E-03	2.96E-05	0.062	0.002	-3.68E-04	3.11E-04	1.85E-03	3.29E-05	0.061	0.002
100	0.25	5	0.25	10	-3.66E-04	1.55E-04	4.83E-04	8.36E-06	0.050	0.002	2.35E-02	1.47E-04	4.31E-04	7.22E-06	0.211	0.003	-2.87E-04	1.54E-04	4.70E-04	7.50E-06	0.053	0.002	-3.66E-04	1.55E-04	4.78E-04	8.31E-06	0.052	0.002
250	0.25	5	0.25	10	9.95E-05	9.78E-05	1.93E-04	3.35E-06	0.049	0.002	2.41E-02	9.22E-05	1.73E-04	2.91E-06	0.456	0.004	1.48E-04	9.69E-05	1.89E-04	3.03E-06	0.050	0.002	9.95E-05	9.78E-05	1.92E-04	3.34E-06	0.050	0.002
750	0.25	5	0.25	10	-9.18E-06	5.71E-05	6.44E-05	1.11E-06	0.053	0.002	2.40E-02	5.38E-05	5.79E-05	9.70E-07	0.884	0.002	1.60E-05	5.66E-05	6.33E-05	1.01E-06	0.053	0.002	-9.18E-06	5.71E-05	6.43E-05	1.11E-06	0.054	0.002
25	0.25	100	0.25	200	2.38E-05	7.50E-05	1.12E-04	8.09E-06	0.050	0.002	1.22E-03	7.48E-05	1.07E-04	7.81E-06	0.062	0.002	2.42E-05	7.50E-05	1.07E-04	7.85E-06	0.061	0.002	2.38E-05	7.50E-05	1.07E-04	7.93E-06	0.060	0.002
100	0.25	100	0.25	200	-8.89E-06	3.76E-05	2.79E-05	1.99E-06	0.051	0.002	1.24E-03	3.75E-05	2.75E-05	1.96E-06	0.059	0.002	-8.41E-06	3.76E-05	2.76E-05	1.97E-06	0.054	0.002	-8.89E-06	3.76E-05	2.76E-05	1.98E-06	0.054	0.002
250	0.25	100	0.25	200	-1.15E-05	2.38E-05	1.12E-05	8.52E-07	0.051	0.002	1.23E-03	2.37E-05	1.11E-05	8.41E-07	0.069	0.002	-1.16E-05	2.38E-05	1.11E-05	8.47E-07	0.052	0.002	-1.15E-05	2.38E-05	1.11E-05	8.50E-07	0.052	0.002
750	0.25	100	0.25	200	6.24E-06	1.36E-05	3.94E-06	4.26E-07	0.050	0.002	1.26E-03	1.36E-05	3.92E-06	4.77E-07	0.099	0.002	6.41E-06	1.36E-05	3.94E-06	4.36E-07	0.050	0.002	6.24E-06	1.36E-05	3.93E-06	4.41E-07	0.050	0.002
25	0.05	5	0.05	10	5.41E-05	1.54E-04	4.90E-04	3.90E-05	0.051	0.002	2.16E-02	9.12E-05	1.90E-04	2.11E-05	0.374	0.003	5.03E-04	1.51E-04	4.85E-04	3.38E-05	0.047	0.001	5.41E-05	1.54E-04	4.70E-04	3.82E-05	0.061	0.002
100	0.05	5	0.05	10	-6.01E-05	7.71E-05	1.22E-04	9.72E-06	0.049	0.002	2.19E-02	4.47E-05	4.62E-05	5.19E-06	0.920	0.002	5.21E-05	7.64E-05	1.21E-04	8.56E-06	0.048	0.002	-6.01E-05	7.71E-05	1.21E-04	9.67E-06	0.051	0.002
250	0.05	5	0.05	10	-5.42E-05	4.93E-05	4.90E-05	3.86E-06	0.049	0.002	2.22E-02	2.84E-05	1.84E-05	2.08E-06	1.000	0.000	-7.90E-07	4.90E-05	4.83E-05	3.43E-06	0.049	0.002	-5.42E-05	4.93E-05	4.88E-05	3.85E-06	0.050	0.002
750	0.05	5	0.05	10	5.39E-05	2.83E-05	1.63E-05	1.31E-06	0.049	0.002	2.22E-02	1.63E-05	6.13E-06	7.84E-07	1.000	0.000	6.76E-05	2.81E-05	1.61E-05	1.17E-06	0.049	0.002	5.39E-05	2.83E-05	1.63E-05	1.31E-06	0.049	0.002
25	0.05	100	0.05	200	-1.58E-06	3.76E-05	2.82E-05	4.79E-06	0.054	0.002	2.09E-03	3.63E-05	2.54E-05	3.98E-06	0.082	0.002	-6.31E-07	3.76E-05	2.72E-05	4.43E-06	0.062	0.002	-1.58E-06	3.76E-05	2.71E-05	4.69E-06	0.064	0.002
100	0.05	100	0.05	200	1.51E-05	1.90E-05	7.07E-06	1.24E-06	0.052	0.002	2.19E-03	1.83E-05	6.56E-06	1.07E-06	0.144	0.002	1.55E-05	1.90E-05	7.01E-06	1.16E-06	0.054	0.002	1.51E-05	1.90E-05	6.99E-06	1.23E-06	0.056	0.002
250	0.05	100	0.05	200	-7.23E-06	1.19E-05	2.94E-06	5.33E-07	0.051	0.002	2.19E-03	1.15E-05	2.77E-06	8.89E-07	0.272	0.003	-6.93E-06	1.19E-05	2.94E-06	4.99E-07	0.052	0.002	-7.23E-06	1.19E-05	2.93E-06	5.56E-07	0.052	0.002
750	0.05	100	0.05	200	-4.05E-06	6.85E-06	1.00E-06	0.00E + 00	0.049	0.002	2.19E-03	6.60E-06	1.00E-06	0.00E + 00	0.647	0.003	-4.04E-06	6.85E-06	1.00E-06	0.00E + 00	0.050	0.002	-4.05E-06	6.85E-06	1.00E-06	0.00E + 00	0.050	0.002

The first 24 rows of the Table describe experiments where the dispersion parameter does not vary as a function of group membership; whereas, in the bottom 24 rows of the Table the dispersion parameter varies as a function of group membership. Rows 1–8 (and 25–32) correspond to simulated experiments where the mass of the distribution is near 0.50, rows 9–16 (and 33–40) correspond to simulated experiments where the mass of the distribution is centered near 0.25, and finally rows 17–24 (and 41–48) correspond to simulated experiments where the mass of the distribution is near 0.05.

∆ = 0 (type-1 error experiments).

Type-1 error refers to proportion of null hypothesis reject (expected 0.05).

**Table 4:** Mean estimates from 20000 replicate simulations of bias (MC error of bias), variance (MC error of variance) and power (MC error power), from the fitted linear, beta, variable-dispersion beta and fractional logit regression models estimated on the beta distributed response data (Power experiments)

					**Linear regression model**						**Beta regression model**						**Variable dispersion beta regression model**						**Fractional logit regression model**					
**N0 = N1**	**μ**_**0**_	**φ**_**0**_	**μ**_**1**_	**φ**_**1**_	**Bias**	**MC error bias**	**Variance**	**MC error variance**	**Power**	**MC error power**	**Bias**	**MC error bias**	**Variance**	**MC error variance**	**Power**	**MC error power**	**Bias**	**MC error bias**	**Variance**	**MC error variance**	**Power**	**MC error power**	**Bias**	**MC error bias**	**Variance**	**MC error variance**	**Power**	**MC error power**
25	0.5	5	0.5	5	4.57E-04	4.07E-04	3.32E-03	3.34E-05	0.071	1.82E-03	4.27E-04	4.03E-04	3.09E-03	2.98E-05	0.088	2.00E-03	4.80E-04	4.04E-04	3.08E-03	2.96E-05	0.090	2.02E-03	4.57E-04	4.07E-04	3.19E-03	3.27E-05	0.085	1.97E-03
100	0.5	5	0.5	5	1.21E-04	2.02E-04	8.33E-04	8.17E-06	0.138	2.44E-03	1.35E-04	2.00E-04	8.05E-04	7.41E-06	0.145	2.49E-03	1.41E-04	2.00E-04	8.04E-04	7.40E-06	0.146	2.50E-03	1.21E-04	2.02E-04	8.24E-04	8.13E-06	0.142	2.47E-03
250	0.5	5	0.5	5	-3.53E-05	1.29E-04	3.33E-04	3.23E-06	0.276	3.16E-03	-3.84E-05	1.27E-04	3.24E-04	2.94E-06	0.284	3.19E-03	-3.46E-05	1.27E-04	3.24E-04	2.94E-06	0.284	3.19E-03	-3.53E-05	1.29E-04	3.32E-04	3.23E-06	0.279	3.17E-03
750	0.5	5	0.5	5	2.32E-05	7.42E-05	1.11E-04	1.09E-06	0.659	3.35E-03	1.90E-05	7.34E-05	1.08E-04	9.95E-07	0.670	3.32E-03	2.07E-05	7.34E-05	1.08E-04	9.95E-07	0.671	3.32E-03	2.32E-05	7.42E-05	1.11E-04	1.09E-06	0.660	3.35E-03
25	0.5	100	0.5	100	1.51E-04	9.91E-05	1.98E-04	1.00E-05	0.416	3.48E-03	1.49E-04	9.91E-05	1.90E-04	9.80E-06	0.451	3.52E-03	1.49E-04	9.91E-05	1.90E-04	9.80E-06	0.451	3.52E-03	1.51E-04	9.91E-05	1.90E-04	9.82E-06	0.451	3.52E-03
100	0.5	100	0.5	100	1.54E-05	5.00E-05	4.95E-05	2.45E-06	0.942	1.66E-03	1.58E-05	4.99E-05	4.90E-05	2.44E-06	0.944	1.62E-03	1.57E-05	5.00E-05	4.90E-05	2.44E-06	0.944	1.62E-03	1.54E-05	5.00E-05	4.90E-05	2.44E-06	0.944	1.62E-03
250	0.5	100	0.5	100	-4.13E-05	3.14E-05	1.98E-05	1.00E-06	1.000	8.66E-05	-4.11E-05	3.14E-05	1.97E-05	9.98E-07	1.000	8.66E-05	-4.11E-05	3.14E-05	1.97E-05	9.98E-07	1.000	8.66E-05	-4.13E-05	3.14E-05	1.97E-05	9.97E-07	1.000	8.66E-05
750	0.5	100	0.5	100	-5.02E-06	1.82E-05	6.65E-06	6.55E-07	1.000	0.00E + 00	-4.95E-06	1.82E-05	6.63E-06	6.62E-07	1.000	0.00E + 00	-5.02E-06	1.82E-05	6.63E-06	6.62E-07	1.000	0.00E + 00	-5.02E-06	1.82E-05	6.63E-06	6.61E-07	1.000	0.00E + 00
25	0.25	5	0.25	5	-4.33E-05	3.57E-04	2.57E-03	3.65E-05	0.076	1.87E-03	-1.47E-04	3.34E-04	2.14E-03	3.04E-05	0.093	2.05E-03	-1.33E-05	3.54E-04	2.40E-03	3.18E-05	0.091	2.03E-03	-4.33E-05	3.57E-04	2.47E-03	3.58E-05	0.090	2.02E-03
100	0.25	5	0.25	5	6.89E-05	1.80E-04	6.45E-04	9.12E-06	0.166	2.63E-03	1.69E-04	1.68E-04	5.54E-04	7.75E-06	0.191	2.78E-03	1.27E-04	1.78E-04	6.24E-04	8.14E-06	0.173	2.68E-03	6.89E-05	1.80E-04	6.39E-04	9.08E-06	0.171	2.66E-03
250	0.25	5	0.25	5	2.85E-05	1.13E-04	2.58E-04	3.58E-06	0.340	3.35E-03	-2.92E-05	1.05E-04	2.23E-04	3.07E-06	0.387	3.44E-03	2.63E-06	1.12E-04	2.51E-04	3.21E-06	0.349	3.37E-03	2.85E-05	1.13E-04	2.57E-04	3.57E-06	0.343	3.36E-03
750	0.25	5	0.25	5	-4.56E-05	6.57E-05	8.60E-05	1.21E-06	0.768	2.98E-03	-6.05E-05	6.13E-05	7.44E-05	1.04E-06	0.826	2.68E-03	-5.12E-05	6.50E-05	8.41E-05	1.09E-06	0.777	2.94E-03	-4.56E-05	6.57E-05	8.59E-05	1.21E-06	0.769	2.98E-03
25	0.25	100	0.25	100	2.72E-04	8.70E-05	1.53E-04	9.03E-06	0.516	3.53E-03	2.80E-04	8.67E-05	1.46E-04	8.69E-06	0.555	3.51E-03	2.73E-04	8.70E-05	1.47E-04	8.76E-06	0.552	3.52E-03	2.72E-04	8.70E-05	1.47E-04	8.85E-06	0.551	3.52E-03
100	0.25	100	0.25	100	3.28E-05	4.39E-05	3.83E-05	2.19E-06	0.981	9.65E-04	3.00E-05	4.38E-05	3.77E-05	2.15E-06	0.982	9.31E-04	3.24E-05	4.39E-05	3.80E-05	2.17E-06	0.982	9.43E-04	3.28E-05	4.39E-05	3.80E-05	2.18E-06	0.982	9.43E-04
250	0.25	100	0.25	100	-6.18E-06	2.78E-05	1.53E-05	9.19E-07	1.000	0.00E + 00	-5.28E-06	2.77E-05	1.52E-05	9.03E-07	1.000	0.00E + 00	-6.03E-06	2.78E-05	1.53E-05	9.12E-07	1.000	0.00E + 00	-6.18E-06	2.78E-05	1.53E-05	9.20E-07	1.000	0.00E + 00
750	0.25	100	0.25	100	2.44E-05	1.60E-05	5.02E-06	2.17E-07	1.000	0.00E + 00	2.49E-05	1.60E-05	5.01E-06	1.65E-07	1.000	0.00E + 00	2.43E-05	1.60E-05	5.01E-06	1.99E-07	1.000	0.00E + 00	2.44E-05	1.60E-05	5.02E-06	2.06E-07	1.000	0.00E + 00
25	0.05	5	0.05	5	3.18E-06	1.97E-04	7.78E-04	4.36E-05	0.147	2.50E-03	-3.96E-05	1.22E-04	2.99E-04	2.63E-05	0.302	3.25E-03	5.81E-05	1.94E-04	7.58E-04	3.78E-05	0.152	2.54E-03	3.18E-06	1.97E-04	7.47E-04	4.27E-05	0.170	2.65E-03
100	0.05	5	0.05	5	-1.26E-04	9.84E-05	1.95E-04	1.08E-05	0.438	3.51E-03	-9.09E-05	6.01E-05	7.28E-05	6.49E-06	0.855	2.49E-03	-1.31E-04	9.72E-05	1.91E-04	9.55E-06	0.447	3.52E-03	-1.26E-04	9.84E-05	1.93E-04	1.08E-05	0.447	3.52E-03
250	0.05	5	0.05	5	5.34E-05	6.24E-05	7.80E-05	4.33E-06	0.812	2.76E-03	4.24E-05	3.81E-05	2.90E-05	2.62E-06	0.998	3.46E-04	5.54E-05	6.18E-05	7.65E-05	3.86E-06	0.821	2.71E-03	5.34E-05	6.24E-05	7.77E-05	4.33E-06	0.814	2.75E-03
750	0.05	5	0.05	5	-1.36E-06	3.61E-05	2.59E-05	1.47E-06	0.999	2.74E-04	-1.36E-05	2.19E-05	9.63E-06	9.35E-07	1.000	0.00E + 00	-1.56E-06	3.57E-05	2.55E-05	1.31E-06	0.999	2.55E-04	-1.36E-06	3.61E-05	2.59E-05	1.47E-06	0.999	2.69E-04
25	0.05	100	0.05	100	4.94E-05	4.79E-05	4.62E-05	5.71E-06	0.952	1.51E-03	5.38E-05	4.64E-05	4.16E-05	4.81E-06	0.970	1.20E-03	4.93E-05	4.79E-05	4.45E-05	5.32E-06	0.960	1.38E-03	4.94E-05	4.79E-05	4.43E-05	5.59E-06	0.960	1.39E-03
100	0.05	100	0.05	100	2.09E-06	2.41E-05	1.16E-05	1.45E-06	1.000	0.00E + 00	3.33E-06	2.33E-05	1.07E-05	1.25E-06	1.000	0.00E + 00	2.01E-06	2.41E-05	1.15E-05	1.36E-06	1.000	0.00E + 00	2.09E-06	2.41E-05	1.15E-05	1.44E-06	1.000	0.00E + 00
250	0.05	100	0.05	100	-2.59E-05	1.51E-05	4.64E-06	8.17E-07	1.000	0.00E + 00	-2.90E-05	1.46E-05	4.25E-06	7.46E-07	1.000	0.00E + 00	-2.60E-05	1.51E-05	4.63E-06	8.10E-07	1.000	0.00E + 00	-2.59E-05	1.51E-05	4.62E-06	8.25E-07	1.000	0.00E + 00
750	0.05	100	0.05	100	1.35E-06	8.74E-06	1.74E-06	1.18E-06	1.000	0.00E + 00	7.73E-07	8.46E-06	1.14E-06	1.16E-06	1.000	0.00E + 00	1.27E-06	8.74E-06	1.74E-06	1.18E-06	1.000	0.00E + 00	1.35E-06	8.74E-06	1.73E-06	1.20E-06	1.000	0.00E + 00
25	0.5	5	0.5	10	-2.57E-04	3.56E-04	2.58E-03	3.14E-05	0.075	1.86E-03	-1.42E-03	3.59E-04	2.47E-03	3.03E-05	0.089	2.01E-03	-1.99E-04	3.54E-04	2.40E-03	2.83E-05	0.091	2.04E-03	-2.57E-04	3.56E-04	2.47E-03	3.08E-05	0.088	2.01E-03
100	0.5	5	0.5	10	-3.96E-05	1.80E-04	6.43E-04	7.72E-06	0.165	2.62E-03	-1.34E-03	1.81E-04	6.39E-04	7.60E-06	0.161	2.60E-03	-7.90E-05	1.78E-04	6.25E-04	7.12E-06	0.173	2.68E-03	-3.96E-05	1.80E-04	6.37E-04	7.69E-06	0.171	2.66E-03
250	0.5	5	0.5	10	-4.50E-05	1.13E-04	2.57E-04	3.05E-06	0.341	3.35E-03	-1.29E-03	1.14E-04	2.57E-04	3.01E-06	0.313	3.28E-03	-3.11E-05	1.12E-04	2.52E-04	2.82E-06	0.348	3.37E-03	-4.50E-05	1.13E-04	2.56E-04	3.04E-06	0.343	3.36E-03
750	0.5	5	0.5	10	4.49E-05	6.56E-05	8.58E-05	1.02E-06	0.770	2.97E-03	-1.22E-03	6.62E-05	8.61E-05	1.01E-06	0.725	3.16E-03	4.43E-05	6.52E-05	8.43E-05	9.50E-07	0.777	2.95E-03	4.49E-05	6.56E-05	8.57E-05	1.02E-06	0.771	2.97E-03
25	0.5	100	0.5	200	-8.21E-06	8.56E-05	1.49E-04	9.09E-06	0.517	3.53E-03	-6.91E-05	8.57E-05	1.43E-04	8.93E-06	0.551	3.52E-03	-8.83E-06	8.56E-05	1.42E-04	8.89E-06	0.554	3.51E-03	-8.21E-06	8.56E-05	1.43E-04	8.91E-06	0.553	3.52E-03
100	0.5	100	0.5	200	2.59E-06	4.32E-05	3.72E-05	2.26E-06	0.984	8.95E-04	-5.88E-05	4.33E-05	3.69E-05	2.26E-06	0.984	8.79E-04	3.25E-06	4.32E-05	3.68E-05	2.25E-06	0.985	8.69E-04	2.59E-06	4.32E-05	3.68E-05	2.25E-06	0.985	8.73E-04
250	0.5	100	0.5	200	-4.29E-06	2.73E-05	1.49E-05	9.32E-07	1.000	0.00E + 00	-6.64E-05	2.74E-05	1.48E-05	9.32E-07	1.000	0.00E + 00	-4.03E-06	2.73E-05	1.48E-05	9.30E-07	1.000	0.00E + 00	-4.29E-06	2.73E-05	1.48E-05	9.30E-07	1.000	0.00E + 00
750	0.5	100	0.5	200	-1.16E-05	1.58E-05	5.00E-06	1.54E-07	1.000	0.00E + 00	-7.40E-05	1.58E-05	5.00E-06	1.56E-07	1.000	0.00E + 00	-1.14E-05	1.58E-05	4.99E-06	1.55E-07	1.000	0.00E + 00	-1.16E-05	1.58E-05	4.99E-06	1.56E-07	1.000	0.00E + 00
25	0.25	5	0.25	10	3.61E-04	3.15E-04	1.97E-03	3.36E-05	0.098	2.10E-03	2.22E-02	2.99E-04	1.73E-03	2.89E-05	0.223	2.94E-03	6.24E-04	3.12E-04	1.85E-03	2.96E-05	0.116	2.27E-03	3.61E-04	3.15E-04	1.89E-03	3.29E-05	0.113	2.24E-03
100	0.25	5	0.25	10	-1.24E-04	1.57E-04	4.94E-04	8.31E-06	0.201	2.83E-03	2.26E-02	1.49E-04	4.48E-04	7.27E-06	0.613	3.44E-03	-5.32E-05	1.56E-04	4.80E-04	7.50E-06	0.211	2.89E-03	-1.24E-04	1.57E-04	4.89E-04	8.26E-06	0.206	2.86E-03
250	0.25	5	0.25	10	2.94E-05	9.96E-05	1.97E-04	3.33E-06	0.430	3.50E-03	2.29E-02	9.42E-05	1.80E-04	2.90E-06	0.945	1.62E-03	6.90E-05	9.89E-05	1.94E-04	3.01E-06	0.439	3.51E-03	2.94E-05	9.96E-05	1.97E-04	3.32E-06	0.433	3.50E-03
750	0.25	5	0.25	10	1.16E-04	5.72E-05	6.59E-05	1.12E-06	0.867	2.40E-03	2.31E-02	5.45E-05	6.02E-05	9.81E-07	1.000	0.00E + 00	1.25E-04	5.67E-05	6.47E-05	1.01E-06	0.874	2.35E-03	1.16E-04	5.72E-05	6.58E-05	1.11E-06	0.867	2.40E-03
25	0.25	100	0.25	200	-1.67E-04	7.55E-05	1.14E-04	8.12E-06	0.625	3.42E-03	9.73E-04	7.53E-05	1.10E-04	7.94E-06	0.695	3.26E-03	-1.66E-04	7.55E-05	1.09E-04	7.89E-06	0.659	3.35E-03	-1.67E-04	7.55E-05	1.09E-04	7.96E-06	0.658	3.36E-03
100	0.25	100	0.25	200	2.74E-05	3.77E-05	2.85E-05	2.00E-06	0.997	4.12E-04	1.20E-03	3.76E-05	2.84E-05	1.99E-06	0.999	2.69E-04	2.76E-05	3.77E-05	2.82E-05	1.98E-06	0.997	3.96E-04	2.74E-05	3.77E-05	2.82E-05	2.00E-06	0.997	3.90E-04
250	0.25	100	0.25	200	1.30E-05	2.40E-05	1.14E-05	8.55E-07	1.000	0.00E + 00	1.20E-03	2.39E-05	1.14E-05	8.51E-07	1.000	0.00E + 00	1.32E-05	2.40E-05	1.14E-05	8.45E-07	1.000	0.00E + 00	1.30E-05	2.40E-05	1.14E-05	8.53E-07	1.000	0.00E + 00
750	0.25	100	0.25	200	1.13E-05	1.38E-05	3.98E-06	2.35E-07	1.000	0.00E + 00	1.20E-03	1.38E-05	3.99E-06	1.98E-07	1.000	0.00E + 00	1.13E-05	1.38E-05	3.98E-06	2.41E-07	1.000	0.00E + 00	1.13E-05	1.38E-05	3.98E-06	2.45E-07	1.000	0.00E + 00
25	0.05	5	0.05	10	-3.00E-05	1.68E-04	5.69E-04	3.79E-05	0.229	2.97E-03	2.24E-02	1.10E-04	2.79E-04	2.19E-05	0.867	2.40E-03	3.93E-04	1.65E-04	5.58E-04	3.26E-05	0.242	3.03E-03	-3.00E-05	1.68E-04	5.46E-04	3.71E-05	0.252	3.07E-03
100	0.05	5	0.05	10	6.17E-05	8.43E-05	1.42E-04	9.39E-06	0.561	3.51E-03	2.32E-02	5.35E-05	6.95E-05	5.41E-06	1.000	0.00E + 00	1.74E-04	8.36E-05	1.40E-04	8.27E-06	0.574	3.50E-03	6.17E-05	8.43E-05	1.41E-04	9.34E-06	0.569	3.50E-03
250	0.05	5	0.05	10	-5.70E-05	5.35E-05	5.69E-05	3.77E-06	0.899	2.13E-03	2.33E-02	3.41E-05	2.78E-05	2.18E-06	1.000	0.00E + 00	-1.86E-05	5.31E-05	5.61E-05	3.34E-06	0.903	2.09E-03	-5.70E-05	5.35E-05	5.66E-05	3.76E-06	0.901	2.11E-03
750	0.05	5	0.05	10	-2.69E-05	3.09E-05	1.90E-05	1.27E-06	1.000	1.00E-04	2.34E-02	1.96E-05	9.28E-06	7.90E-07	1.000	0.00E + 00	-1.14E-05	3.06E-05	1.87E-05	1.14E-06	1.000	8.66E-05	-2.69E-05	3.09E-05	1.90E-05	1.27E-06	1.000	1.00E-04
25	0.05	100	0.05	200	-1.85E-05	4.07E-05	3.27E-05	4.82E-06	0.984	8.91E-04	2.03E-03	3.96E-05	3.18E-05	4.38E-06	0.997	4.06E-04	-1.74E-05	4.06E-05	3.15E-05	4.49E-06	0.988	7.76E-04	-1.85E-05	4.07E-05	3.14E-05	4.73E-06	0.987	7.92E-04
100	0.05	100	0.05	200	1.15E-05	2.02E-05	8.15E-06	1.23E-06	1.000	0.00E + 00	2.14E-03	1.96E-05	8.17E-06	1.14E-06	1.000	0.00E + 00	1.21E-05	2.02E-05	8.08E-06	1.16E-06	1.000	0.00E + 00	1.15E-05	2.02E-05	8.07E-06	1.23E-06	1.000	0.00E + 00
250	0.05	100	0.05	200	-1.34E-05	1.28E-05	3.16E-06	7.35E-07	1.000	0.00E + 00	2.13E-03	1.25E-05	3.17E-06	7.53E-07	1.000	0.00E + 00	-1.32E-05	1.28E-05	3.14E-06	6.89E-07	1.000	0.00E + 00	-1.34E-05	1.28E-05	3.15E-06	7.11E-07	1.000	0.00E + 00
750	0.05	100	0.05	200	9.87E-06	7.31E-06	1.00E-06	0.00E + 00	1.000	0.00E + 00	2.16E-03	7.09E-06	1.00E-06	0.00E + 00	1.000	0.00E + 00	9.78E-06	7.31E-06	1.00E-06	0.00E + 00	1.000	0.00E + 00	9.87E-06	7.31E-06	1.00E-06	0.00E + 00	1.000	0.00E + 00

The first 24 rows of the Table describe experiments where the dispersion parameter does not vary as a function of group membership; whereas, in the bottom 24 rows of the Table the dispersion parameter varies as a function of group membership. Rows 1–8 (and 25–32) correspond to simulated experiments where the mass of the distribution is near 0.50, rows 9–16 (and 33–40) correspond to simulated experiments where the mass of the distribution is centered near 0.25, and finally rows 17–24 (and 41–48) correspond to simulated experiments where the mass of the distribution is near 0.05.

∆ = 0.025 (power experiments).

Power refers to the proportion of null hypothesis rejected.

**Table 5:** Mean estimates from 20000 replicate simulations of bias (MC error of bias), variance (MC error of variance) and type-1 error (MC error type-1 error), from the fitted linear, beta, variable-dispersion beta and fractional logit regression models estimated on the multinomial distributed response data (Type-1 error experiments)

			**Linear regression model**						**Beta regression model**						**Variable dispersion beta regression model**						**Fractional logit regression model**					
**N0 = N1**	**E(Y**_**0**_**)**	**E(Y**_**1**_**)**	**Bias**	**MC error bias**	**Variance**	**MC error variance**	**Type-1 Error**	**MC Error Type-1 Error**	**Bias**	**MC error bias**	**Variance**	**MC error variance**	**Type-1 Error**	**MC Error Type-1 Error**	**Bias**	**MC error bias**	**Variance**	**MC error variance**	**Type-1 Error**	**MC Error Type-1 Error**	**Bias**	**MC error bias**	**Variance**	**MC error variance**	**Type-1 Error**	**MC Error Type-1 Error**
25	0.5	0.5	-6.22E-04	2.64E-04	1.40E-03	3.04E-05	0.048	0.002	-6.48E-04	2.72E-04	1.39E-03	3.04E-05	0.062	0.002	-6.43E-04	2.71E-04	1.38E-03	3.02E-05	0.063	0.002	-6.22E-04	2.64E-04	1.34E-03	2.98E-05	0.060	0.002
100	0.5	0.5	8.00E-06	1.32E-04	3.50E-04	7.51E-06	0.049	0.002	-2.97E-06	1.37E-04	3.61E-04	7.68E-06	0.055	0.002	-4.54E-07	1.37E-04	3.61E-04	7.66E-06	0.055	0.002	8.00E-06	1.32E-04	3.46E-04	7.47E-06	0.052	0.002
250	0.5	0.5	1.07E-04	8.33E-05	1.40E-04	3.02E-06	0.051	0.002	1.09E-04	8.60E-05	1.46E-04	3.10E-06	0.053	0.002	1.09E-04	8.59E-05	1.45E-04	3.10E-06	0.053	0.002	1.07E-04	8.33E-05	1.39E-04	3.01E-06	0.052	0.002
750	0.5	0.5	-3.01E-06	4.84E-05	4.67E-05	1.01E-06	0.051	0.002	-2.56E-06	4.99E-05	4.87E-05	1.03E-06	0.054	0.002	-2.57E-06	4.99E-05	4.87E-05	1.03E-06	0.054	0.002	-3.01E-06	4.84E-05	4.66E-05	1.01E-06	0.052	0.002
25	0.215	0.215	-4.46E-04	3.72E-04	2.74E-03	3.41E-05	0.051	0.002	-3.06E-04	2.80E-04	1.82E-03	3.12E-05	0.037	0.001	-3.94E-04	3.51E-04	2.19E-03	3.13E-05	0.072	0.002	-4.46E-04	3.72E-04	2.63E-03	3.34E-05	0.062	0.002
100	0.215	0.215	5.06E-05	1.85E-04	6.86E-04	8.38E-06	0.050	0.002	-2.96E-05	1.38E-04	4.64E-04	7.95E-06	0.030	0.001	5.99E-05	1.74E-04	5.63E-04	7.95E-06	0.061	0.002	5.06E-05	1.85E-04	6.79E-04	8.33E-06	0.053	0.002
250	0.215	0.215	1.18E-04	1.17E-04	2.74E-04	3.31E-06	0.051	0.002	1.19E-04	8.69E-05	1.86E-04	3.17E-06	0.030	0.001	1.08E-04	1.10E-04	2.26E-04	3.16E-06	0.060	0.002	1.18E-04	1.17E-04	2.73E-04	3.30E-06	0.053	0.002
750	0.215	0.215	-1.10E-05	6.78E-05	9.13E-05	1.11E-06	0.050	0.002	-1.93E-05	5.02E-05	6.20E-05	1.06E-06	0.029	0.001	-1.64E-06	6.37E-05	7.55E-05	1.06E-06	0.059	0.002	-1.10E-05	6.78E-05	9.12E-05	1.11E-06	0.050	0.002

Response variables were generated from a discrete multinomial distribution with probability mass observed only on points in (0,1). Multinomial response probabilities for this experiment are given in Table 2 above.

∆ = 0 (type-1 error experiments).

Type-1 error refers to the proportion of null hypothesis rejected (expected 0.05).

**Table 6:** Mean estimates from 20000 replicate simulations of bias (MC error of bias), variance (MC Error Variance), and Power (MC Error Power), from the fitted linear, beta, variable-dispersion beta and fractional logit regression models estimated on the multinomial distributed response data (Power experiments)

			**Linear regression model**						**Beta regression model**						**Variable dispersion beta regression model**						**Fractional logit regression model**					
**N0 = N1**	**E(Y**_**0**_**)**	**E(Y**_**1**_**)**	**Bias**	**MC error bias**	**Variance**	**MC error variance**	**Power**	**MC error power**	**Bias**	**MC error bias**	**Variance**	**MC error variance**	**Power**	**MC error power**	**Bias**	**MC error bias**	**Variance**	**MC error variance**	**Power**	**MC error power**	**Bias**	**MC error bias**	**Variance**	**MC error variance**	**Power**	**MC error power**
25	0.5	0.6	-1.36E-04	2.63E-04	1.40E-03	3.04E-05	0.745	0.003	2.32E-03	2.76E-04	1.44E-03	3.22E-05	0.769	0.003	1.77E-03	2.73E-04	1.43E-03	3.20E-05	0.767	0.003	-1.36E-04	2.63E-04	1.35E-03	2.98E-05	0.774	0.003
100	0.5	0.6	-2.17E-04	1.33E-04	3.50E-04	7.54E-06	1.000	0.000	2.37E-03	1.39E-04	3.72E-04	8.18E-06	1.000	0.000	1.86E-03	1.38E-04	3.72E-04	8.16E-06	1.000	0.000	-2.17E-04	1.33E-04	3.46E-04	7.50E-06	1.000	0.000
250	0.5	0.6	-5.06E-05	8.32E-05	1.40E-04	3.02E-06	1.000	0.000	2.55E-03	8.73E-05	1.50E-04	3.29E-06	1.000	0.000	2.05E-03	8.64E-05	1.50E-04	3.28E-06	1.000	0.000	-5.06E-05	8.32E-05	1.39E-04	3.01E-06	1.000	0.000
750	0.5	0.6	-8.70E-05	4.85E-05	4.66E-05	1.01E-06	1.000	0.000	2.55E-03	5.08E-05	5.02E-05	1.10E-06	1.000	0.000	2.05E-03	5.04E-05	5.02E-05	1.10E-06	1.000	0.000	-8.70E-05	4.85E-05	4.66E-05	1.01E-06	1.000	0.000
25	0.215	0.315	2.82E-04	3.70E-04	2.75E-03	3.42E-05	0.466	0.004	1.35E-02	2.96E-04	2.02E-03	3.04E-05	0.725	0.003	3.33E-03	3.55E-04	2.21E-03	3.07E-05	0.589	0.003	2.82E-04	3.70E-04	2.64E-03	3.35E-05	0.501	0.004
100	0.215	0.315	6.30E-06	1.84E-04	6.85E-04	8.36E-06	0.966	0.001	1.36E-02	1.46E-04	5.17E-04	7.65E-06	1.000	0.000	3.10E-03	1.77E-04	5.68E-04	7.71E-06	0.987	0.001	6.30E-06	1.84E-04	6.78E-04	8.32E-06	0.967	0.001
250	0.215	0.315	1.87E-05	1.17E-04	2.74E-04	3.31E-06	1.000	0.000	1.37E-02	9.28E-05	2.08E-04	3.04E-06	1.000	0.000	3.14E-03	1.12E-04	2.28E-04	3.07E-06	1.000	0.000	1.87E-05	1.17E-04	2.73E-04	3.30E-06	1.000	0.000
750	0.215	0.315	1.02E-04	6.74E-05	9.14E-05	1.10E-06	1.000	0.000	1.38E-02	5.33E-05	6.94E-05	1.02E-06	1.000	0.000	3.22E-03	6.45E-05	7.64E-05	1.03E-06	1.000	0.000	1.02E-04	6.74E-05	9.13E-05	1.11E-06	1.000	0.000

Response variables were generated from a discrete multinomial distribution with probability mass observed only on points in (0,1). Multinomial response probabilities for this experiment are given in Table 2 above.

∆ = 0.10 (power experiments).

Power refers to the proportion of null hypothesis rejected.

Table 3 describes the results of the type-1 error experiment (∆ = 0) given response data distributed according to independent draws from a beta distribution. The top half of Table 3 illustrates results when the dispersion parameter is equal across groups; whereas, the bottom half of Table 3 illustrates results when the dispersion parameter varies as a function of group membership. As probability mass moves away from the center of the support (i.e. 0.5) and towards the boundary of the support (0 or 1) we observe that the beta regression model provides biased estimates of the average proportion/percentage/rate difference between the two samples when the dispersion parameters vary as a function of group membership. For example, when μ_0_ = μ_1_ = 0.25 and *ϕ*_0_ = 5 and *ϕ*_1_ = 10 we observe biased estimates of effect from the beta regression model (biases range from 2.27E-02 through 2.41E-02). As the dispersion parameters increase (i.e. *ϕ*_0_ = 100 and *ϕ*_1_ = 200) the observed bias in the beta regression model is slightly attenuated (biases range from 1.22E-03 through 1.26E-03). Similar findings are observed when the mean parameters are adjusted, such that μ_0_ = μ_1_ = 0.05. Table 4 describes the results of the power experiments (∆ = 0.025) given response data distributed according to independent draws from a beta distribution. Near identical results are observed as were discussed for the type-1 error experiments in Table 3. That is, when the respective means are near the boundary of the support, and the dispersion parameters vary as a function of group membership the beta regression model can yield biased estimates of effect. When the dispersion parameters are small (*ϕ*_0_ = 5 and *ϕ*_1_ = 10) the bias in effect estimates is appreciable (biases range from 2.22E-02 through 2.31E-02). On an absolute scale these biases are meaningful; however, when expressed on a relative scale these biases are even more pronounced. As the dispersion parameters increase in magnitude the magnitude of the bias in the beta regression models is attenuated (biases range from 9.73E-04 through 1.20E-03). Given that the simple beta regression model is biased in certain scenarios we eliminate it from consideration in the results/discussion sections which follow.

In small sample scenarios, when N_0_ = N_1_ = 25, the linear regression model had a mean type-1 error of approximately 0.050; whereas, the variable-dispersion beta regression model had mean type-1 error rate of 0.058 and the fractional logit regression model had a mean type-1 error rate of 0.060. As the sample size is increased to 100 per group, 250 per group and 750 per group, respectively, the type-1 error rates of the linear regression model, the variable dispersion beta regression model and the fractional logit regression model became more similar. Further, improvements in the type-1 error rate of the variable-dispersion beta regression model for small samples (N_0_ = N_1_ = 25) were observed when we used bias corrected/reduced estimation methods instead of the more traditional ML estimation methods (results not shown; however, can be verified by modifying simulation codes in R).

When considering the power experiments estimates of average bias across the 20,000 replicate experiments were small for the linear regression model, the variable-dispersion beta regression model and the fractional logit regression model (of magnitude 1E-04 through 1E-06 respectively). Further, estimates of average variance across the 20,000 replicate simulations were similar across the linear regression model, the variable-dispersion beta regression model and the fractional logit regression model. These findings imply the estimators have similar average mean squared error. That said, the power for estimated variable-dispersion beta regression models and the fractional logit regression models, respectively, marginally exceeded that of the linear regression model across all simulation experiments considered.

Table 5 describes the results of the type-1 error experiment (∆ = 0) given response data distributed according to independent draws from a multinomial distribution. For the type-1 error experiments all estimators are relatively free of bias. The magnitudes of estimated biases are similar for the linear regression model, variable-dispersion beta regression model and the fractional logit model. Again, average variance across the 20,000 replicate simulations were similar for all models. Type-1 error rates are closest to the desired 5% level for the linear regression model. Again the variable-dispersion beta regression model and the fractional logit regression model have elevated type-1 error rates when sample sizes are small (N_0_ = N_1_ = 25). Table 6 describes the results of the power experiment (∆ = 0.10) given response data distributed according to independent draws from a multinomial distribution. When data are simulated according to either a symmetric or asymmetric discrete multinomial distribution we observe that the beta regression model is biased. In the symmetric case biases are attenuated (biases range from 2.32E-03 through 2.55E-03) compared to the asymmetric case (biases range from 1.35E-02 through 1.38E-02). The magnitude of the bias in the linear regression estimator and the fractional logit regression estimator are similar. However, in the case of discrete data we notice that the variable-dispersion beta regression model has slightly elevated mean bias levels. That said, the variable-dispersion beta regression model is slightly more powerful than the linear regression model and the fractional logit regression model (however, this is likely an artifact of the difference in magnitudes of bias in these models). Among models with comparable biases, the fractional logit model is more powerful than the linear regression model when data are generated from a discrete multinomial distribution on (0,1).

## Discussion

The main findings of this Monte Carlo simulation study are summarized in Tables 3, 4, 5 and 6 in the results section. In general, properties of the respective estimators are similar regardless of whether the underlying data generating mechanism is beta distributed (Tables 3 and 4) or multinomial distributed (Tables 5 and 6). Hence we will discuss findings from the type-1 error experiments and the power experiments in general, as results seem to hold irrespective of the probability generating models. We note interesting exceptions where warranted.

Considering the type-1 error experiments (Table 3 and Table 5) we observe that the linear regression model, the variable-dispersion beta regression model and the fractional logit regression model provide unbiased estimates of our population proportion/percentage/rate difference (∆ = 0) under all simulated scenarios. The magnitudes of bias tend to be similar across estimators, ranging from 1E-04 through 1E-06. In many circumstances the simple beta regression model also provide unbiased estimates of our null (∆ = 0) effect. However, in circumstances where the dispersion parameter varied between groups, the simple beta regression model demonstrated fairly substantial bias in its attempt to recover the average population proportion/percentage/rate difference. The impact of non-constant dispersion amongst individuals in this simulation experiment were more pronounced when the dispersion parameters were small (e.g. *ϕ*_0_ = 5 and *ϕ*_1_ = 10) compared to when the dispersion parameters were large (e.g. *ϕ*_0_ = 100 and *ϕ*_1_ = 200). Further, the effects of non-constant dispersion between groups appear more pronounced when the group means are near the boundary of the distributions support (0 or 1) compared to when they are near the center of the support (½). This is demonstrated by observed biases in the beta regression model of about 0.02 units in certain circumstances (Table 3). It is interesting to note that in terms of type-1 error rates the linear regression model performed well regardless of sample size; whereas, the variable-dispersion beta regression model and fractional logit regression model experienced slightly elevated type-1 error rates when the group specific sample sizes were small (N_0_ = N_1_ = 25). Another important point is that improvements in the small sample type-1 error rates of the beta regression estimators could be achieved by using the bias corrected/reduced estimation methods in place of the more traditional ML estimators. These BC/BR estimators are easily implemented in the R betareg() procedure [12,13].

Considering the power experiments (Table 4 and Table 6) we again observe that the linear regression model, the variable-dispersion beta regression model and the fractional logit regression model provide (relatively) unbiased estimates of our proportion/percentage/rate difference (∆ = 0.025 in the beta distributed simulations and ∆ = 0.10 in the multinomial distributed simulations). The magnitude of the average biases across the 20,000 replicate experiments is similar across these three models when the data are beta distributed (Table 4); however, when the data arise from a multinomial distribution the variable-dispersion beta regression model has slightly elevated bias levels compared to the linear regression model and fractional logit regression model. That said, on a relative (or absolute) scale, the observed biases in the variable-dispersion beta regression model are not overly large. Again, in cases where the dispersion parameter varies across groups we observe that the simple beta regression model has trouble recovering the desired epidemiological effect measure. Again, this problem is more pronounced when the dispersion parameters are small and the group means are situated near the boundary of the support. The beta regression model also struggles at recovering the desired difference measure in the multinomial experiment where the response variable is skewed (Table 6); however, demonstrates more comparable performance to the linear regression model, the variable-dispersion beta regression model and the fractional logit model when the response variable is simulated from a symmetric multinomial distribution. In general, the linear regression model, the variable-dispersion beta regression model and the fractional logit regression model perform well in terms of recovering unbiased estimates of the non-zero effect measure. The models have similar power profiles across the continuous beta distributed simulation experiments – with minor power advantages appearing in the variable-dispersion beta regression models and the fractional logit regression models. Further when response data are distributed according to a discrete multinomial distribution minor advantages in power appear for the variable-dispersion beta regression model (at the cost of small magnitude increases in bias) and the fractional logit model compared to the linear regression model (Table 6).

The results of this Monte Carlo simulation study indicate that the linear regression model, the variable-dispersion beta regression model and the fractional logit regression model are capable of producing unbiased estimates of average proportion/percentage/rate differences given response data observed on the interval (0,1) from a two sample design. The simple beta regression model struggles if the dispersion sub-model is incorrectly specified. When sample sizes are small, type-1 error rates appear closer to the nominal 5% level in the linear regression model. The variable-dispersion beta regression model and fractional logit model appear slightly more powerful than the linear regression model when a non-zero difference between groups is present.

A similar study was conducted by Kieschnick and McCullough [21]. In their article they made similar conclusions favouring the (variable-dispersion) beta regression model and the fractional logit regression model for estimating covariate effects on response data observed on (0,1). In their article they dismissed the linear regression model, because in the more complex regression scenarios they were considering it could lead to inadmissible predictions (e.g. predicted values outside of (0,1)). In our simulation experiment we are not necessarily interested in the predictions or fitted values, rather we are interested in the ability of our model to recover the average difference in proportions/percentages/rates across a two-sample design. This is typically viewed as an absolute measure of “effect” in epidemiological research. If one is interested in this measure of effect, rather than in model based predictions then it appears from this simulation that the linear regression model performs similarly well as the more novel variable-dispersion beta regression model and the fractional logit model. That said, if ones interest lies in predicted/fitted-values then it likely behoves the researcher to choose a model which will result in admissible predictions.

Introduction of the beta distribution into a general regression framework has resulted in enhanced attention/use of the beta distribution by both theoretical and applied researchers. Ferrari [7] provides many examples in which the beta regression model has been applied in theoretical/applied modelling exercises. Two biomedical applications where the beta regression framework has been implemented include modelling scales scores, such as SF-6D response data [22] and modelling stroke lesion volumes [23]. Many other biomedical applications of the beta regression model exist as suggested by Ferrari [7].

The purpose of this Monte Carlo simulation experiment was to investigate the properties of the linear regression model, the beta regression model, the variable-dispersion beta regression model and the fractional logit regression model at recovering average proportion/percentage/rate differences from a two sample design. The simplicity of the design aids in interpreting properties of the respective models. In addition, the two sample design is one of the most commonly encountered study designs by epidemiologists and biostatisticians. Hence the simulation study answers a very important question for applied epidemiologists/biostatisticians, namely: given a more traditional linear regression framework for modelling covariate effects on response data observed on the interval (0,1) are there any benefits in fitting a more novel beta regression model, variable-dispersion beta regression model or fractional logit regression model to these same data and using it for inference? Results of this simulation study suggest that the more novel variable-dispersion beta regression model and fractional logit regression model have comparable properties to the traditional linear regression model. While the linear regression model may perform better in terms of type-1 error rates in small samples, the variable-dispersion beta regression model and fractional logit regression model seem slightly more powerful at detecting a true non-zero difference between groups in a two-sample design. Conversely, the simple beta regression model appears to struggle if the dispersion sub-model is incorrectly specified. Given this finding applied researchers should be cautious in fitting off the shelf beta regression models to their (0,1) response data. If a choice is made to fit a beta regression model to observed data practitioners must strive to ensure correct specification of both the mean and dispersion sub-models in order to generate proper inferences.

## Conclusion

The purpose of this Monte Carlo simulation study was to compare the properties of the linear regression model to the more novel beta regression, variable-dispersion beta regression and fractional logit regression models at recovering estimates of average proportion/percentage/rate differences in a two-sample design. We observe that the simple beta regression model is biased if the dispersion sub-model is incorrectly specified. The variable-dispersion beta regression model is unbiased (given proper specification of the dispersion sub-model). The fractional logit regression model is also an unbiased estimator of effect. Moreover, the power and type-1 error profiles are very similar for the linear model, variable-dispersion beta regression model and the fractional logit regression model. These results seem to suggest promise for the beta regression model going forward; however, for the time being applied researchers should be cautious in applying off the shelf beta regression algorithms to their response data observed on the interval (0,1) and should strive to ensure correct specification of both the mean and dispersion sub-models such that proper inferences are generated from the observed data.

## Competing interest

Neither of the two authors of this manuscript have any competing interest to disclose.

## Authors’ contributions

CM played a role in the conceptualization of the study, programming the simulation, interpreting results and writing the first draft of the manuscript. RM played a role in the conceptualization of the study, programming the simulation, interpreting the results and critically revising drafts of the manuscript. Both authors read and approved the final manuscript.

## Pre-publication history

The pre-publication history for this paper can be accessed here:

http://www.biomedcentral.com/1471-2288/14/14/prepub
