# Erratum to: A Monte Carlo simulation study comparing linear regression, beta regression, variable-dispersion beta regression and fractional logit regression at recovering average difference measures in a two sample design

**DOI:** 10.1186/s12874-016-0256-6

**Published:** 2016-11-09

**Authors:** Christopher Meaney, Rahim Moineddin

**Affiliations:** Department of Family and Community Medicine, University of Toronto, 500 University Avenue, Toronto, M5G1V7 ON Canada

## Erratum

After publication of the original article [1], the authors noticed an error in Fig. 1. The legend included in the original sub-plot of Fig. 1 was labelled “phi = 500 (*p =* 25, q = 475)”; however, the figure title suggested phi = 1000.

**Fig. 1:**
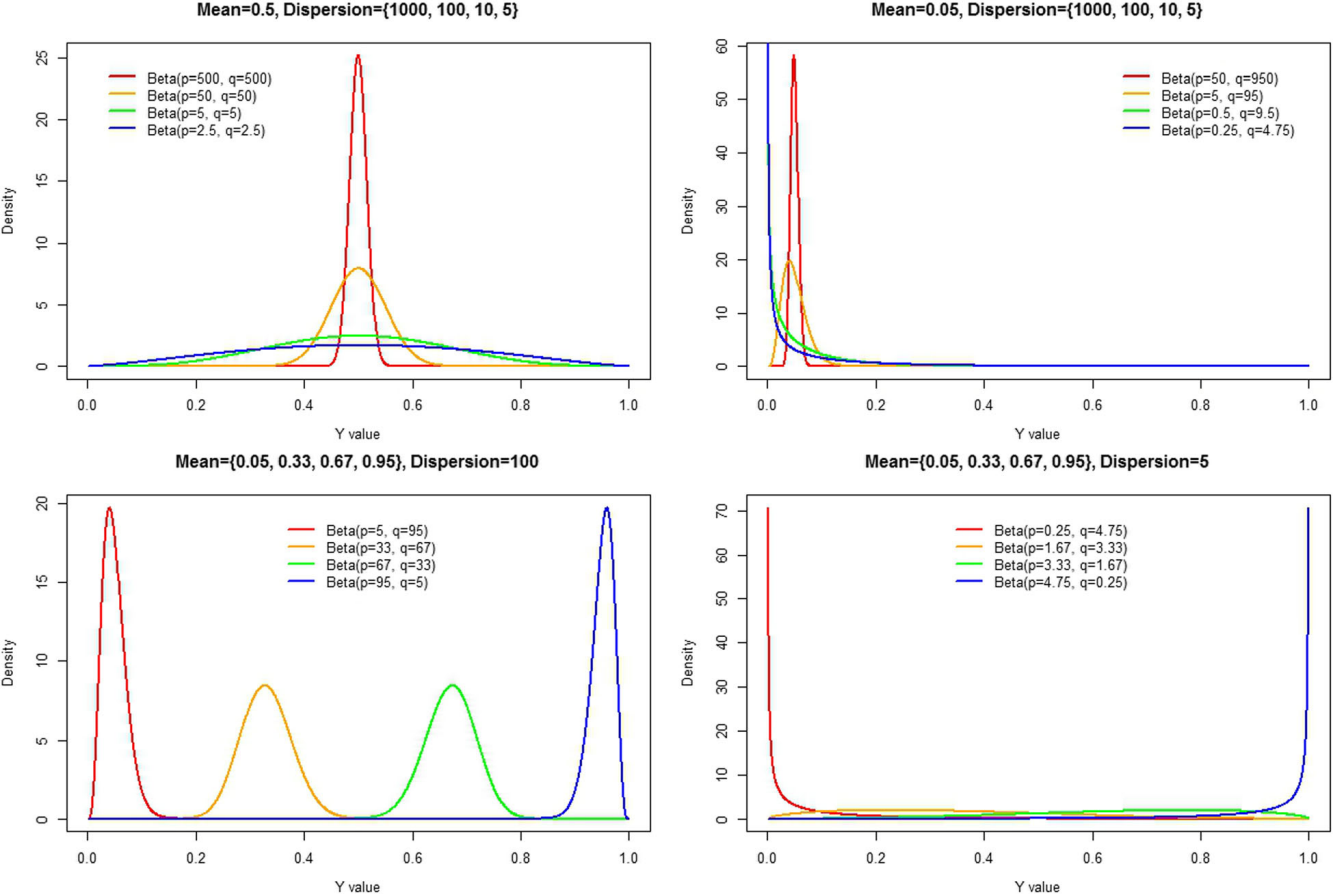
Various forms of the beta density for varying shape parameters {p,q}. Top left panel: We fix the mean equal to 0.5 and plot the resulting beta densities for varying dispersion parameters. Top right panel: We fix the mean equal to 0.05 and plot the resulting beta densities for varying dispersion parameters. Bottom left panel: We fix the dispersion parameter equal to 100 and plot the resulting beta densities for varying mean parameters. Bottom right panel: We fix the dispersion parameter equal to 5 and plot the resulting beta densities for varying mean parameters

An updated version of Fig. 1 is published in this erratum, where the legend has been updated to “phi = 1000 (*p =* 50, q = 950)” to be consistent with the figure title.
